# Supplementary material for: Allosteric regulation and crystallographic fragment screening of SARS-CoV-2 NSP15 endoribonuclease
Source: Nucleic Acids Res. 2023 Apr 28;51(10):5255–70. doi: 10.1093/nar/gkad314 (PMC10250223; doi:10.1093/nar/gkad314)
Supplement: gkad314_Supplemental_Files [file gkad314_supplemental_files.zip › Supplementary datasets.zip!/231/Refine_8/01-BUSTER/Cycle-2/shell.01/R_Rfree.html]

# Rxpct factors in resolution bins at cycle 0


|  |  |  |  |  |  |  |  |  |
| --- | --- | --- | --- | --- | --- | --- | --- | --- |
|  | **<d> (Å)**  Rxpct\_Work | Rxpct\_Free | Rxpct\_All | |  |  |  |  |
|  | 13.1576 | 0.3392 | 0.3904 | 0.3414 |  |
|  | 7.5965 | 0.2964 | 0.3095 | 0.2972 |  |
|  | 5.8843 | 0.2871 | 0.2740 | 0.2865 |  |
|  | 4.9731 | 0.1936 | 0.2010 | 0.1940 |  |
|  | 4.3859 | 0.1645 | 0.1495 | 0.1637 |  |
|  | 3.9672 | 0.1813 | 0.1831 | 0.1814 |  |
|  | 3.6493 | 0.2051 | 0.2263 | 0.2062 |  |
|  | 3.3973 | 0.2138 | 0.2211 | 0.2142 |  |
|  | 3.1912 | 0.2007 | 0.2107 | 0.2012 |  |
|  | 3.0186 | 0.1950 | 0.2033 | 0.1954 |  |
|  | 2.8712 | 0.1995 | 0.1932 | 0.1992 |  |
|  | 2.7435 | 0.1894 | 0.1951 | 0.1897 |  |
|  | 2.6315 | 0.1879 | 0.2132 | 0.1894 |  |
|  | 2.5322 | 0.1918 | 0.1772 | 0.1909 |  |
|  | 2.4433 | 0.1950 | 0.1896 | 0.1947 |  |
|  | 2.3632 | 0.2006 | 0.2077 | 0.2009 |  |
|  | 2.2904 | 0.2006 | 0.1939 | 0.2003 |  |
|  | 2.2240 | 0.2091 | 0.2172 | 0.2095 |  |
|  | 2.1631 | 0.2119 | 0.2319 | 0.2129 |  |
|  | 2.1069 | 0.2558 | 0.2470 | 0.2553 |  |
|  | **Overall** | 0.2135 | 0.2177 | 0.2137 |  |

# Rxpct factors in resolution bins at cycle 1


|  |  |  |  |  |  |  |  |  |
| --- | --- | --- | --- | --- | --- | --- | --- | --- |
|  | **<d> (Å)**  Rxpct\_Work | Rxpct\_Free | Rxpct\_All | |  |  |  |  |
|  | 13.1576 | 0.3394 | 0.3913 | 0.3416 |  |
|  | 7.5965 | 0.2971 | 0.3109 | 0.2979 |  |
|  | 5.8843 | 0.2871 | 0.2733 | 0.2866 |  |
|  | 4.9731 | 0.1949 | 0.2013 | 0.1952 |  |
|  | 4.3859 | 0.1662 | 0.1499 | 0.1653 |  |
|  | 3.9672 | 0.1822 | 0.1852 | 0.1823 |  |
|  | 3.6493 | 0.2065 | 0.2289 | 0.2076 |  |
|  | 3.3973 | 0.2147 | 0.2231 | 0.2151 |  |
|  | 3.1912 | 0.2043 | 0.2113 | 0.2047 |  |
|  | 3.0186 | 0.1989 | 0.2088 | 0.1993 |  |
|  | 2.8712 | 0.2035 | 0.1987 | 0.2033 |  |
|  | 2.7435 | 0.1931 | 0.1987 | 0.1934 |  |
|  | 2.6315 | 0.1909 | 0.2148 | 0.1923 |  |
|  | 2.5322 | 0.1926 | 0.1763 | 0.1916 |  |
|  | 2.4433 | 0.1945 | 0.1870 | 0.1941 |  |
|  | 2.3632 | 0.1994 | 0.2067 | 0.1998 |  |
|  | 2.2904 | 0.1999 | 0.1923 | 0.1995 |  |
|  | 2.2240 | 0.2079 | 0.2171 | 0.2083 |  |
|  | 2.1631 | 0.2117 | 0.2321 | 0.2126 |  |
|  | 2.1069 | 0.2567 | 0.2467 | 0.2561 |  |
|  | **Overall** | 0.2149 | 0.2190 | 0.2151 |  |

# Rxpct factors in resolution bins at cycle 2


|  |  |  |  |  |  |  |  |  |
| --- | --- | --- | --- | --- | --- | --- | --- | --- |
|  | **<d> (Å)**  Rxpct\_Work | Rxpct\_Free | Rxpct\_All | |  |  |  |  |
|  | 13.1576 | 0.3395 | 0.3924 | 0.3418 |  |
|  | 7.5965 | 0.2975 | 0.3115 | 0.2983 |  |
|  | 5.8843 | 0.2872 | 0.2729 | 0.2867 |  |
|  | 4.9731 | 0.1959 | 0.2023 | 0.1963 |  |
|  | 4.3859 | 0.1675 | 0.1508 | 0.1666 |  |
|  | 3.9672 | 0.1830 | 0.1865 | 0.1832 |  |
|  | 3.6493 | 0.2075 | 0.2299 | 0.2086 |  |
|  | 3.3973 | 0.2153 | 0.2244 | 0.2158 |  |
|  | 3.1912 | 0.2059 | 0.2114 | 0.2062 |  |
|  | 3.0186 | 0.2003 | 0.2121 | 0.2008 |  |
|  | 2.8712 | 0.2045 | 0.2001 | 0.2043 |  |
|  | 2.7435 | 0.1941 | 0.1988 | 0.1943 |  |
|  | 2.6315 | 0.1914 | 0.2150 | 0.1928 |  |
|  | 2.5322 | 0.1924 | 0.1769 | 0.1915 |  |
|  | 2.4433 | 0.1935 | 0.1868 | 0.1932 |  |
|  | 2.3632 | 0.1985 | 0.2052 | 0.1988 |  |
|  | 2.2904 | 0.1990 | 0.1907 | 0.1986 |  |
|  | 2.2240 | 0.2074 | 0.2164 | 0.2078 |  |
|  | 2.1631 | 0.2111 | 0.2324 | 0.2121 |  |
|  | 2.1069 | 0.2561 | 0.2461 | 0.2555 |  |
|  | **Overall** | 0.2155 | 0.2198 | 0.2158 |  |

# Rxpct factors in resolution bins at cycle 3


|  |  |  |  |  |  |  |  |  |
| --- | --- | --- | --- | --- | --- | --- | --- | --- |
|  | **<d> (Å)**  Rxpct\_Work | Rxpct\_Free | Rxpct\_All | |  |  |  |  |
|  | 13.1576 | 0.3278 | 0.3742 | 0.3298 |  |
|  | 7.5965 | 0.2760 | 0.3093 | 0.2780 |  |
|  | 5.8843 | 0.3008 | 0.3170 | 0.3014 |  |
|  | 4.9731 | 0.2243 | 0.2628 | 0.2265 |  |
|  | 4.3859 | 0.2081 | 0.2004 | 0.2076 |  |
|  | 3.9672 | 0.2143 | 0.2306 | 0.2151 |  |
|  | 3.6493 | 0.2314 | 0.2728 | 0.2334 |  |
|  | 3.3973 | 0.2345 | 0.2664 | 0.2362 |  |
|  | 3.1912 | 0.2331 | 0.2274 | 0.2328 |  |
|  | 3.0186 | 0.2222 | 0.2287 | 0.2225 |  |
|  | 2.8712 | 0.2209 | 0.2275 | 0.2212 |  |
|  | 2.7435 | 0.2082 | 0.2299 | 0.2092 |  |
|  | 2.6315 | 0.2019 | 0.2309 | 0.2036 |  |
|  | 2.5322 | 0.1911 | 0.1921 | 0.1912 |  |
|  | 2.4433 | 0.1838 | 0.1896 | 0.1841 |  |
|  | 2.3632 | 0.1849 | 0.1858 | 0.1850 |  |
|  | 2.2904 | 0.1815 | 0.1775 | 0.1813 |  |
|  | 2.2240 | 0.1945 | 0.2114 | 0.1953 |  |
|  | 2.1631 | 0.1957 | 0.2177 | 0.1968 |  |
|  | 2.1069 | 0.2402 | 0.2237 | 0.2393 |  |
|  | **Overall** | 0.2301 | 0.2471 | 0.2310 |  |
